# Application of starter cultures in the production of *Enturire* – a traditional sorghum‐based alcoholic beverage

**DOI:** 10.1002/fsn3.438

**Published:** 2016-10-25

**Authors:** Ivan M. Mukisa, Denis Ntaate, Stellah Byakika

**Affiliations:** ^1^ Department of Food Technology and NutritionSchool of Food Technology Nutrition and BioengineeringCollege of Agricultural and Environmental SciencesMakerere UniversityKampalaUganda

**Keywords:** *Enturire*, lactic acid bacteria, *Lactobacillus plantarum*, *obushera*, *Saccharomyces cerevi*siae, Sorghum

## Abstract

*Enturire* is an alcoholic sorghum‐ and honey‐based beverage traditionally produced by spontaneous fermentation. Its fermentation process is lengthy (5–7 days), does not guarantee product quality and safety and thus necessitates use of pure starter cultures. This study compared a modified production process of *Enturire*, with honey added at the start to the traditional one (honey added 3 days into the fermentation). The study also evaluated two starter culture combinations (*L. plantarum* MNC 21 +  *S. cerevisiae* MNC 21 Y) and (*L. plantarum* MNC 21 +  *W. confusa* MNC 20 +  *S. cerevisiae* MNC 21Y). Microbial counts, pH, alcohol content, titratable acidity, (TA) and consumer acceptability of the *Enturire* were determined. Lactic acid bacteria (LAB) and yeast counts increased from 4–6 log cfu/ml to 8–9 log cfu/ml and 3–4 log cfu/ml to 5–8 log cfu/ml, respectively. Acidification of *Enturire* to pH <4.5 was significantly (*p *<* *.05) faster (12 hr) with starter fermentations than in the modified (18 hr) and the traditional processes (31 hr). More alcohol (9.2%–9.4%) was produced by the starters than the spontaneous fermentations (3.24%–4.38%). The modified process without starters produced a more acceptable (*p *< .05) product than the traditional process. The starters produced acceptable *Enturire* within 12 hr with pH, acidity, and alcohol content of about 3.8, 0.7%, and 2.5%–3.5%. Both starter combinations can thus be used to produce safe and acceptable *Enturire* in a short time.

## 1 Introduction

Traditional fermented foods have socioeconomic and nutrition roles they play in developing countries. They are an important part of the local diet and are vital for promoting the cultural heritage of different communities. Some fermented foods are used in various festivals such as births, circumcision, marriage, and burial ceremonies (Lyumugabe, Gros, Nzungize, Bajyana, & Thonart, 2012). Most of these foods are a source of income for many households. These foods also contribute to energy and protein intake (Lyumugabe et al., 2012). Fermentation also generally improves sensory qualities, nutritive value, shelf life, and safety of food products (Arici & Daglioglu, 2002; Mukisa, 2012; Muyanja, Narvhus, Treimo, & Langsrud, 2003). Traditional fermented foods are generally classified into acidic, alkaline, alcoholic, or mixed acidic‐alcoholic products (Steinkraus, 2004).

Traditional alcoholic beverages from Africa include: *Boza* (Arici & Daglioglu, 2002), *Pito* beer (Orji, Mbata, Aniche, & Ahonkhai, 2003), *Togwa* (Mugula, Narvhus, & Sørhaug, 2003), *Dolo* beer (Glover, Sawadogo‐Lingani, Diawara, Jespersen, & Jakobsen, 2009), *Kunu‐zaki* (Agarry, Nkama, & Akoma, 2010), *Ikigage* (Lyumugabe et al., 2012), and the *Enturire* type of *Obushera* (Mukisa et al., 2010; Muyanja, Kikafunda, Narvhus, Helgetun, & Langsrud, 2003). These beverages are characteristically sour (pH = 3.1–4.0) and alcoholic (1%–7%) since their fermentation is dominated by lactic acid bacteria (LAB) and yeasts. Traditional alcoholic fermented beverages are generally produced under uncontrolled and unhygienic conditions which compromise their quality and safety (Achi, 2005). Their production processes are often labor intensive with low productivity which limits large‐scale industrial production. These beverages could become extinct if measures are not taken to improve their appeal, shelf life, homogeneity, ease of handling, and processing.

*Enturire* is a sweet and sour alcoholic sorghum‐ and honey‐based beverage with its origins in south western Uganda (Mukisa et al., 2010). It belongs to a group of naturally fermented sorghum and or millet beverages collectively known as *Obushera*. *Enturire* is produced by fermenting a sorghum malt slurry for about 3 days to yield a sour beverage called *Obutoko* (Mukisa et al., 2010). Honey is then added to *Obutoko* and this is fermented for a further 2–4 days to produce *Enturire*. The resultant product has a pH, acidity, total soluble solids and alcohol content of 4.2–3.9, 0.84%–1.33% lactic acid, 9.7–12.9 °Brix, and 1.9%–6%, respectively (Mukisa, 2012).

Processing of *Enturire* relies on spontaneous fermentation in which undefined cultures containing both the desirable and nondesirable flora can grow in the product (Lyumugabe et al., 2012; Mukisa, Porcellato, et al., 2012). Inconsistencies in microbiota development contribute to compromising the esthetic quality and safety of the product (Lyumugabe et al., 2012; Mukisa, Porcellato, et al., 2012). Application of starters, especially for industrial processes, can hasten fermentation, facilitate improved process control as well as ensure consistent product quality and enhance safety (Holzapfel, 2002; Leroy & De Vuyst, 2004). These advantages have been demonstrated in studies on traditional fermented products such as *Kenkey* (Halm, Osei‐Yaw, Hayford, Kpodo, & Amoa‐Awua, 1996)*, Uji* (Masha, Ipsen, Petersen, & Jakobsen, 1998)*, Pito* beer (Orji et al., 2003), *Togwa* (Mugula et al., 2003), *Mageu* (Holzapfel & Taljaard, 2004), *Dolo* beer (Glover et al., 2009), *Kunu‐zaki* (Agarry et al., 2010), and *ikigage* (Lyumugabe, Uyisenga, Songa, & Thonart, 2014).

LAB and yeasts dominating in *Obushera* fermentation have been evaluated for their potential use as starters for the production of *Obutoko* type of *Obushera* (Mukisa, 2012; Muyanja, Narvhus, & Langsrud, 2012). Combinations of *S. cerevisiae* with *L. plantarum* and either *W. confusa* or *Lactococcus lactis* produce a profile of flavor compounds similar to that of traditionally produced *Obushera* (Mukisa, 2012). The products were, however, not subjected to sensory evaluation. These starters have also not been evaluated for production of *Enturire*. The fermentation of *Enturire* is also longer than that of the other types of *Obushera* by 2–4 days since it involves addition of honey after 3 days and fermenting the product further. This process can be modified or shortened with minimal effect on final product characteristics. It was, however, unknown whether modifying the production process of *Enturire* by adding honey at the start of the fermentation and using starter cultures could yield acceptable *Enturire* in a shorter time.

Therefore, the aim of this study was to evaluate the effect of modifying the fermentation process of *Enturire,* by adding honey at the start and using starter cultures, on the duration of the fermentation and acceptability of the final product. Use of starters to produce acceptable *Enturire* in a shorter time can help significantly boost the commercial industrial production of *Enturire*.

## 2 Materials and Methods

### 2.1 Sorghum malt flour

Sorghum grains (Eyera variety) for preparing sorghum malt were obtained from the National Semi‐ arid Resources Research Center (NaSARRI) in Serere, Uganda. Sorghum grains were placed on a raised wire mesh and sorted to remove chaff. The grain was then washed using potable pressurized water. Clean grain was then soaked in potable water containing 0.2% sodium hydroxide for 24 hr to remove tannins and to soften the grains so as to ease germination. The grain was rinsed in potable water, spread on metallic trays and left to germinate in a germination chamber for 2 days at ambient temperature. Germination was stopped by drying the grain in a cabinet drier at 60°C for 24 hr to achieve a moisture content of 14%. The malt was then ground into flour using a wonder mill (Grote Molen Inc., Pocatello, USA) and kept in air‐tight containers.

### 2.2 Honey

Black wattle honey (dark colored) obtained from Arua district in Uganda was purchased from Green and White Enterprises Ntinda, Kampala. The honey was stored in a plastic container at ambient temperature.

### 2.3 Microbial strains

#### 2.3.1 *Saccharomyces cerevisiae* MNC 21Y

*Saccharomyces cerevisiae* MNC 21Y, previously isolated from *Obushera* (Mukisa, Porcellato, et al., 2012) and stored at −40°C in quarter strength Ringer's solution containing 15% glycerol was used. The yeast was propagated according to the procedure described by Mukisa (2012). From the stock, 0.1 ml was inoculated into 100 ml of sterile Yeast Mold Broth (Laboratorios, CONDA, Madrid, Spain) and incubated at 30°C for 72 hr. The yeast was subcultured thrice after which the cells were separated from the broth (centrifuging at 7,500*g* for 10 min at 4°C) and washed with sterile quarter strength Ringer's solution. The resulting pellet were resuspended in 10 ml of sterile quarter strength Ringer's solution to produce a stock containing 7.0 log cfu/mL of *S. cerevisiae* MNC 21Y.

#### 2.3.2 *Lactobacillus plantarum* MNC 21 and *Weissela confusa* MNC 20

LAB starters (*L. plantarum* MNC 21 and *W. confusa* MNC 20) previously isolated from *Obushera* (Mukisa, Porcellato, et al., 2012)*,* and stored at −40°C in quarter strength Ringer's solution containing 15% glycerol were used. The LAB were singly propagated according to the procedure described by Mukisa (2012). From the stock, 0.1 ml was inoculated in 100 ml of sterile MRS broth (Laboratorios, CONDA, Madrid, Spain) and incubated at 30°C for 24 hr. Each LAB was subcultured thrice after which cells were separated from the broth (centrifuging at 7,500*g* for 10 min at 4°C) and washed with sterile quarter strength Ringer's solution. The resulting pellets of each LAB were separately resuspended in 10 ml of sterile quarter strength Ringer's solution to produce a stock containing 9.8 log cfu/ml of *L. plantarum* MNC 21 and 9.5 log cfu/ml of *W. confusa* MNC 20.

### 2.4 Production of *Enturire*

**Figure 1:**
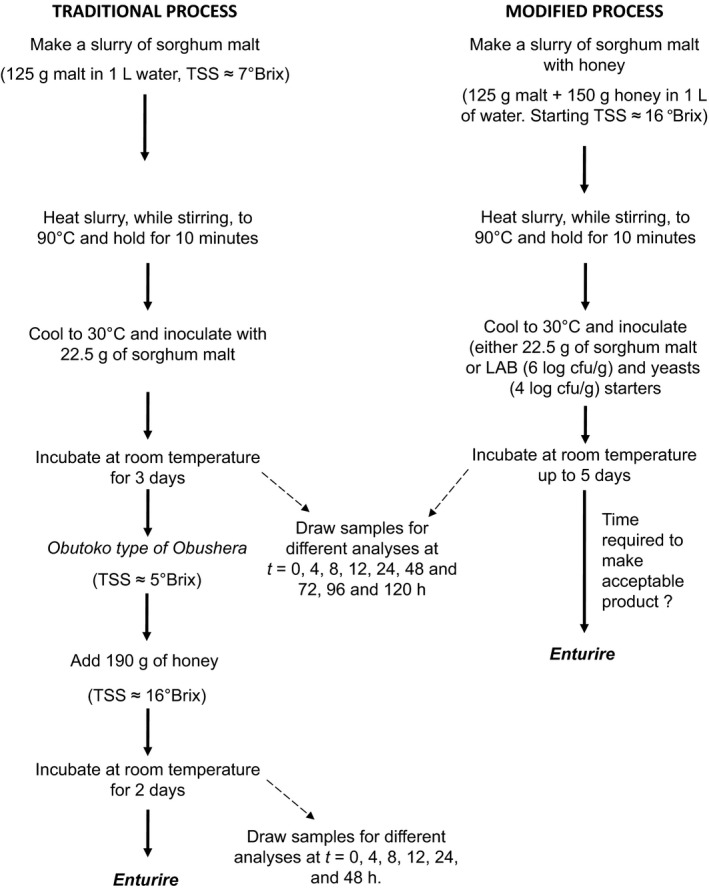
The traditional process used for spontaneous fermentation of an alcoholic sorghum‐ and honey‐based beverage (*Enturire*) (Mukisa et al., 2010) and a modified process with honey added at the start followed by either a spontaneous fermentation or fermentation with starter cultures

*Enturire* was produced by following two major approaches as shown in Figure 1. First was the traditional spontaneous fermentation (Mukisa et al., 2010) in which 125 g of malted sorghum flour were added to 1 L of potable water to make a slurry. The slurry was heated in a batch pasteurizer with constant stirring to 90°C and held at this temperature for 10 min. The mixture was cooled to 30°C and inoculated with 22.5 g of sorghum malt to initiate fermentation. Fermentation was carried out at room temperature (25°C) for 3 days to produce *Obutoko*. The *Obutoko* was then sweetened with 190 g of honey and left to ferment further for 2 days to produce *Enturire*. The second procedure involved modifying the traditional process. In this procedure, honey was added at the start of the process and the amount adjusted so as to bring the total soluble solids to 16°Brix. This value is equivalent to the soluble solids content at the point when honey is added in the traditional process. Initiation of fermentation was done as in the traditional procedure (using sorghum malt) or by use of starter cultures (*L. plantarum* + *S. cerevisiae*;* L. plantarum* + *W. confusa* + *S. cerevisiae*). The LAB were inoculated at a concentration of 6 log cfu/ml and yeast at 4 log cfu/ml. Fermentation was carried at room temperature (25°C) for 5 days. Microbial counts, pH, titratable acidity (TA), and alcohol content were determined at predetermined time intervals during the fermentation. Sensory acceptability of *Enturire* was also determined at days 4 and 5 of fermentation for fermentations without the starters and at 12 and 24 hr for fermentations with starters. The times for sensory evaluation were selected basing on the attainment of the following characteristics of *Enturire* as reported by Mukisa (2012): pH (4.2–3.9), acidity (0.84%–1.33%), total soluble solids (9.7–12.9 °Brix), and alcohol content (1.9%–6%) of *Enturire*.

### 2.5 Analyses

#### 2.5.1 Microbiological analyses

LAB counts were determined by pour plating selected serial dilutions of *Enturire* in sterile MRS agar (Laboratorios, CONDA, Madrid, Spain) and incubating at 30°C for 48 hr. Yeast counts were determined by surface spreading selected serial dilutions of *Enturire* on sterile prepoured acidified Potato Dextrose Agar (Laboratorios, CONDA, Madrid, Spain) and incubating at 30°C for 72 hr.

#### 2.5.2 Physico‐chemical analyses

The pH was determined using a digital pH meter (pH 98107, USA). The total soluble solids (°Brix at room temperature) were determined using a refractometer (KH0294067, USA). Titratable acidity was determined by titrating 10 mL of the sample against a standardized solution of 0.1mol/L NaOH with phenolphthalein as the indicator (AOAC, 1990). The alcohol content was determined as a percentage by volume using specific gravity tables after distillation (AOAC, 1995).

#### 2.5.3 Sensory evaluation

An untrained consumer panel (*n* = 60) was used to determine the consumer acceptability of *Enturire*. Sensory evaluation was carried out in individual booths. The panelists ranked acceptability of various attributes of different *Enturire* samples using a nine‐point hedonic scale (9 = like extremely, 8 =  like very much, 7 =  like moderately, 6 =  like slightly, 5 =  neither like nor dislike, 4 =  dislike slightly, 3 =  dislike moderately, 2 =  dislike very much, 1 =  dislike extremely).

#### 2.5.4 Statistical analyses

All fermentation experiments were carried out in triplicate. The data were analyzed using the one‐way Analysis of Variance (ANOVA) to test for significant differences (*p* < .05). Mean comparisons were made using the Least Significant Difference (LSD). All statistical analyses were performed using XLSTAT software (version 2010.5.02, Addinsoft, France).

## 3 Results

### 3.1 Microbial counts

Table 1 shows the changes in microbial counts during the production of *Enturire* using the traditional and modified fermentation processes with and without starter cultures. Generally, LAB and yeast counts increased from 4–6 cfu/ml to 8–9 log cfu/ml and 3–4 log cfu/ml to 5–7 log cfu/ml at the point when pH dropped to pH ≤4.5, respectively. Modifying the process by adding honey at the start accelerated the growth rate of microorganisms in *Enturire* with LAB and yeasts counts attained in 24 hr similar to those at 48 hr in the traditional process. Application of starter cultures in the modified process enabled attainment of similar or even higher LAB and yeast counts in a much shorter time (12 hr) compared to the spontaneous fermentation processes (24–48 hr).

**Table 1:** Changes in microbial counts during the traditional and modified fermentation processes of *Enturire*

Process and starters	Microbial counts (Log cfu/ml)				Time (hr) at pH ≤4.5
	LAB		Yeasts		
	(T = 0 hr)	(pH ≤4.5)	(T = 0 hr)	(pH ≤4.5)	
Traditional–Spontaneous	4.5 ± 0.0	8.3 ± 0.0	3.0 ± 0.0	5.1 ± 0.0	31
Modified–Spontaneous	4.3 ± 0.0	8.1 ± 0.0	2.8 ± 0.1	4.9 ± 0.0	18
Modified–MNC 21 + MNC 21Y	6.2 ± 0.0	8.5 ± 0.0	4.2 ± 0.0	6.2 ± 0.0	12
Modified–MNC 20 + MNC 21 + MNC 21Y	6.4 ± 0.0	9.2 ± 0.0	4.2 ± 0.0	7.5 ± 0.0	12

LAB, lactic acid bacteria.

Values are means ± standard deviations of three independent fermentations. Starter cultures are MNC 20: *W. confusa*; MNC 21: *L. plantarum* and MNC 21Y: *S. cerevisiae*.

### 3.2 pH and acidity

**Figure 2:**
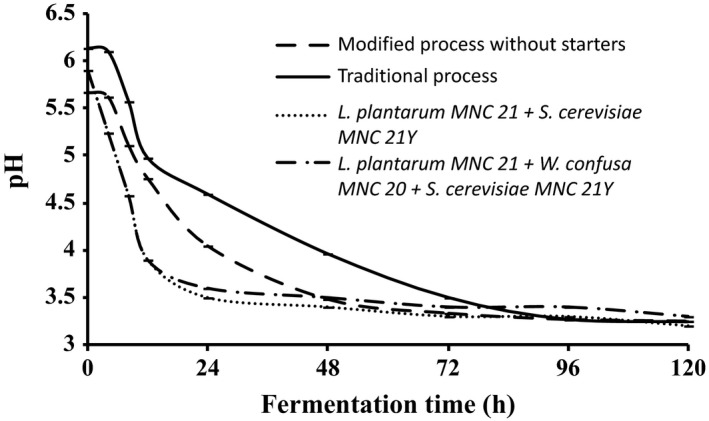
Changes in pH during fermentation of *Enturire* at 25°C using the traditional process, a modified process with honey added at the start and a modified process with pure starter cultures. Error bars show standard deviations of three independent fermentations

**Figure 3:**
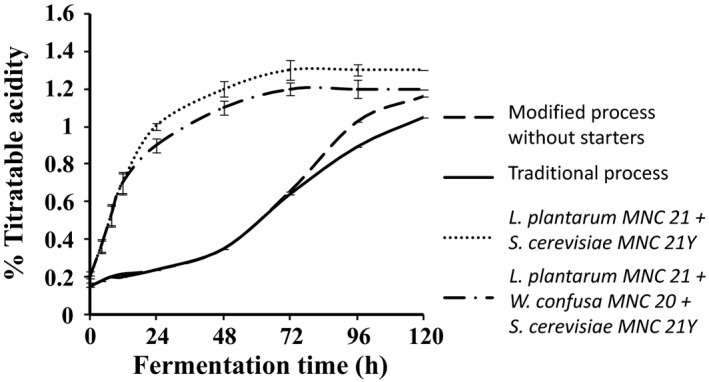
Changes in % titratable acidity during fermentation of *Enturire* at 25°C using the traditional process, a modified process with honey added at the start and a modified process with pure starter cultures. Error bars show standard deviations of three independent fermentations

Changes in pH and titratable acidity during the production of *Enturire* using the traditional and modified fermentation processes are shown in Figures 2 and 3, respectively. The pH of *Enturire* dropped from 5.7–6.0 to 3.2 within 96–120 hr (Figure 2), whereas acidity increased from 0.12%–0.15% to a maximum of 1.0%–1.3% in 48–120 hr (Figure 3). Modifying the process by adding honey at the start accelerated the acidification rate (pH dropped to ≤4.5 in 18 hr compared to 31 hr in the traditional process) and resulted in significantly (*p* < .05) higher titratable acid levels at the end of the fermentation. Application of starter cultures in the modified process significantly (*p* < .05) accelerated the acidification rate (pH ≤ 4.5 attained within 12 hr). Starter fermented *Enturire* attained acidity values ≥0.7% within 12 hr, whereas spontaneously fermented samples took about 72 hr.

### 3.3 Alcohol content

**Figure 4:**
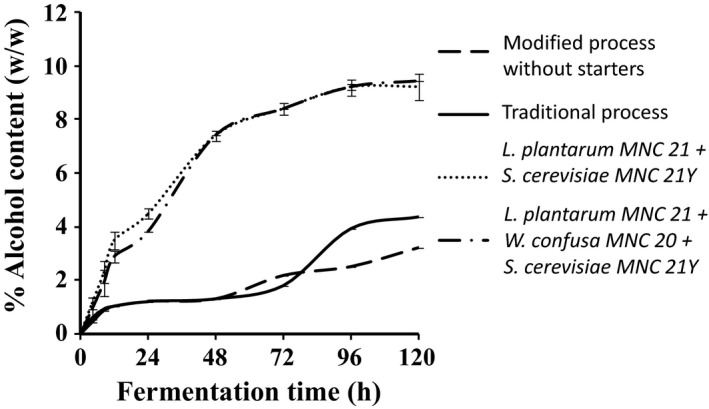
Changes in % alcohol content during fermentation of *Enturire* at 25°C using the traditional process, a modified process with honey added at the start and a modified process with pure starter cultures. Error bars show standard deviations of three independent fermentations

Figure 4 shows the changes in concentrations of alcohol content during the production of *Enturire* using the traditional and modified fermentation processes. Alcohol content increased from 0.0% to a maximum of 3.24%–4.38% and 9.2%–9.4% in spontaneously and starter fermented samples, respectively. Modifying the process by adding honey at the start did not have a significant effect (*p* > .05) on alcohol production during the first 48 hr but significantly (*p* < .05) reduced the final concentration of alcohol. Addition of starter cultures significantly (*p* < .05) increased the rate of alcohol production with alcohol contents of 2.9%–3.5% attained in about 12 hr compared to values of about 1% obtained in the spontaneously fermented samples. By comparison, alcohol contents of >2.0% were only attained after 72 hr in the spontaneously fermented *Enturire*.

### 3.4 Consumer acceptability of *Enturire* produced without starters

Table 2 shows the consumer acceptability scores of *Enturire* produced using the traditional and modified process without the additional of pure starters. *Enturire* produced using both processes was also more acceptable on day 4 than day 5. Modifying the process by adding honey at the start of the process significantly (*p* < .05) increased the acceptability scores of *Enturire*.

**Table 2:** Consumer acceptability scores of *Enturire* produced using the modified and traditional fermentation processes without starters

Processing method	Duration of Fermentation (days)	Acceptability scores				
		Appearance	Taste	Aroma	Mouth feel	Overall acceptability
Modified	4	6.4 ± 1.9^a^	7.2 ± 1.3^a^	6.4 ± 1.9^a^	7.2 ± 1.0^a^	7.3 ± 1.3^a^
Traditional	4	6.5 ± 1.8^a^	5.9 ± 2.0^b^	5.8 ± 2.0^a^	6.1 ± 2.2^b^	6.3 ± 1.9^b^
Modified	5	5.6 ± 2.2^b^	5.1 ± 2.1^c^	5.8 ± 2.2^a^	5.6 ± 2.2^b^	5.6 ± 2.3^b^
Traditional	5	6.3 ± 2.0^ab^	3.9 ± 2.1^d^	5.0 ± 2.2^b^	4.0 ± 2.2^c^	4.5 ± 2.4^c^

Values are means± standard deviations of three independent fermentations. Values in the same column with similar superscripts are not significantly different (*p* > .05). The interpretation of the anchors on the nine‐point hedonic scale used are as follows: 9 = like extremely, 8 =  like very much, 7 =  like moderately, 6 =  like slightly, 5 =  neither like nor dislike, 4 =  dislike slightly, 3 =  dislike moderately, 2 =  dislike very much, 1 =  dislike extremely.

The consumer acceptability scores of *Enturire* made using the modified fermentation process with starter cultures are shown in table 3. Both starter combinations (*L. plantarum* MNC 21 +  *S. cerevisiae* MNC 21Y and *L. plantarum* MNC 21 +  *W. confusa* MNC 20 +  *S. cerevisiae* MNC 21Y) produced acceptable *Enturire* after 12 and 24 hr of fermentation. However, *Enturire* fermented for 12 hr using the *L. plantarum* MNC 21 +  *W. confusa* MNC 20 +  *S. cerevisiae* MNC 21Y starter combination had lowest (*p* < .05) acceptability scores for aroma, taste, mouthfeel, and overall acceptability.

**Table 3:** Consumer acceptability scores of *Enturire* made using the modified fermentation process with pure starter cultures

Starter cultures	Acceptability scores					
	Duration of Fermentation (hr)	Appearance	Taste	Aroma	Mouth feel	Overall acceptability
MNC 21 *+* MNC 21Y	12	6.7 ± 1.5^ab^	6.4 ± 1.6^a^	6.0 ± 1.8^a^	6.8 ± 1.3^a^	6.6 ± 1.6^a^
MNC 20 *+* MNC 21 *+* MNC 21Y	12	6.3 ± 1.9^b^	5.5 ± 2.3^b^	4.7 ± 2.2^b^	5.8 ± 2.0^b^	5.4 ± 2.2^b^
MNC 21 *+* MNC 21Y	24	6.7 ± 1.5^ab^	6.4 ± 1.8^a^	6.0 ± 2.1^a^	6.5 ± 1.7^a^	6.5 ± 1.9^a^
MNC 20 *+* MNC 21 *+* MNC 21Y	24	5.5 ± 2.4^c^	6.4 ± 2.0^a^	6.4 ± 1.7^a^	6.6 ± 1.5^a^	6.6 ± 1.6^a^

Values are means ± standard deviations of three independent fermentations. Values in the same column with similar superscripts are not significantly different (*p* > .05). Starter cultures are MNC 20: *W. confusa*; MNC 21: *L. plantarum* MNC 21 and MNC 21Y: *S. cerevisiae*. The interpretation of the anchors on the nine‐point hedonic scale used are as follows: 9 = like extremely, 8 =  like very much, 7 =  like moderately, 6 =  like slightly, 5 =  neither like nor dislike, 4 =  dislike slightly, 3 =  dislike moderately, 2 =  dislike very much, 1 =  dislike extremely.

## 4 Discussion

The purpose of this study was to evaluate the effect of modifying the fermentation process of *Enturire,* by adding honey at the start and using starter cultures, on the duration of the fermentation and acceptability of the final product. Some of the key factors considered in evaluating the modified process were that traditionally produced *Enturire* has attributes of: pH = 4.2–3.9, 0.84%–1.33% lactic acid, and 1.9%–6% alcohol content (Mukisa, Muyanja, Byaruhanga, Langsrud, & Narvhus, 2012). Additionally, some studies recommend that ensuring safety of lactic acid fermented beverages requires a rapid pH drop to ≤4.5 (Kunene, Hastings, & Von Holy, 1999; Nout, 1992; Nout, Rombouts, & Havelaar, 1989), whereas Steinkraus (1995) recommends attainment of a pH < 4.0 and a titratable acidity of about 0.7% lactic acid. Fast growth of starters would be necessary for realizing rapid acidification.

The counts of LAB and yeasts in spontaneously fermented *Enturire* were in agreement with earlier reports for different types of *Obushera* (Kateu, 1998; Mukisa, Porcellato, et al., 2012; Muyanja, Narvhus, et al., 2003). Application of pure starter cultures allows for better control of the process in that the inoculum concentration can be increased thus enabling the acceleration of the fermentation process. This partly explains why starter fermented samples achieved higher microbial counts in a shorter time compared to the spontaneous fermentation.

Faster growth of LAB and yeasts observed in the modified spontaneous process could be attributed to the inclusion of honey at the start of the fermentation. Honey is a good source fructose (35%–40%) and glucose (30%–39%) and also contains maltose (1.9%–9.8%) all of which are readily fermentable sugars (Shin & Ustunol, 2005). The starting total sugar levels in *Obutoko,* which is the base for *Enturire* fermentation are about 90.9–26.9 g kg^−1^ and these drop to 16.6–<5.0 g kg^−1^ within two to 3 days of fermentation (Mukisa, 2012). Addition of honey to initiate the secondary fermentation that produces *Enturire* increases the total sugars to about 134 g kg^−1^ (Mukisa, 2012). Therefore, modifying the traditional process by adding honey at the start of the fermentation provides almost 1.5–5 times the initial sugar concentrations observed in traditional process. The high initial sugar concentrations thus support faster growth and acidification enabling the desired pH drop (pH 4.5 to <4.0) in 18 hr (Figure 2).

The high initial concentrations of sugars in the modified process, coupled with a higher inoculum in the starter fermented samples further facilitated a much faster acidification (desired pH attained in 12 hr). Faster acidification with application of starter cultures of LAB including *L. plantarum, L. fermentum. L. lactis* has been reported in a number of studies on fermented cereal beverages (Agarry et al., 2010; Halm et al., 1996; Masha et al., 1998; Mugula et al., 2003).

The alcohol content obtained in spontaneously fermented *Enturire* was in the range reported for this product (Mukisa, Muyanja, et al., 2012). Modifying the traditional process by adding honey at the start of fermentation had no effect on ethanol production in the first 72 hr probably because yeast generally grow much slower than LAB. Maximum yeast counts in *Obushera* are generally observed at least 6 hr after those of LAB (Mukisa, 2012). However, after 72 hr, significantly lower amounts of ethanol were produced in the modified process (Figure 4). This could be attributed to competition for fermentable sugars in the earlier stages of fermentation (0–72 hr). The fast growing LAB used most of the sugars for acid production thus explaining a significantly higher yield of acid in the modified spontaneous fermentation than in the traditional process (Figure 3). Furthermore, in the traditional process, honey was added at 72 hr of fermentation and it is at about this time that the LAB, apart from the acid tolerant such as (*L. plantarum*) (Byakika, 2015; Mukisa, 2012) are inhibited by low pH (pH = 3.5) (Figure 2). Acidic conditions generally favor the growth of yeasts. This coupled with the additional fermentable sugars from the honey also explains the rapid increase in alcohol content of the *Enturire* fermented using the traditional process after 72 hr.

The maximum alcohol content of *Enturire* produced with starter cultures (9.2%–9.4%) was higher than that previously reported (1.9%–6%) for *Enturire* (Mukisa, Muyanja, et al., 2012). This could be due to use of pure starter cultures in this work while the results reported in literature were obtained from spontaneously fermented *Enturire*. Spontaneous fermentations are often slow, uncontrolled and are characterized by a series of microbial successions, competitions, and inhibitions. In the case of addition of starters, significantly higher amounts of alcohol were produced (Figure 4) in a shorter time because actively growing starters were introduced moreover at higher concentrations compared to the spontaneous fermentation processes.

*Enturire* produced using the modified process was more acceptable than that produced using the traditional process. Addition of honey prefermentation increased the level of fermentable sugars in the *Enturire* made using the modified process. This could have resulted in higher flavor notes due to production of acetaldehyde and alcohols (Mukisa, 2012; Muyanja et al., 2012) thus the higher acceptability scores. In the *Enturire* made using the traditional method the honey was added 3 days into the fermentation therefore, by the time the sensory analysis was done (on days 4 and 5) the levels of the fruity aromas were probably much lower than in the modified process.

Combinations of *S. cerevisiae* with *L. plantarum* and either *W. confusa* or *Lactococcus lactis,* produce a flavor profile close to that of traditionally processed *Obushera* (Mukisa, 2012). *W. confusa* MNC 20 being a heterofermenter would contribute to flavor of *Obushera* by producing acetic acid (Mukisa, 2012). However, the inclusion of *W. confusa* in the *L. plantarum* MNC 21 *+*  *S. cerevisiae* MNC 21 Y starter combination did not significantly affect overall acceptability. This could be attributed to *W. confusa* MNC 20 being acid sensitive (Mukisa, 2012) thus being inhibited by the increasing acidity of *Enturire* during fermentation.

The production of *Enturire* using the traditional method typically takes 5–7 days depending on how successful the natural fermentation is (Mukisa et al., 2010). In this study it was possible to use the modified process without starters to obtain acceptable *Enturire* within 4 days. This product was more acceptable than that produced using the traditional method. It is also worth noting that *Enturire* with pH ≤4.5, acidity ≥0.7%, and alcohol content ≥2.0% can be produced in 48–72 hr using the modified process without starters. This could therefore shorten the fermentation process by 1–3 days. The application of starters in the modified process can further reduce the fermentation process by up to 12 hr.

## 5 Conclusions

Modifying traditional fermentation processes by eliminating some stages or reordering some process steps and applying starters may offer opportunities for shortening their processing. With respect to *Enturire,* modifying the traditional production process by adding honey pre rather than midfermentation produces a more acceptable product moreover in a shorter time compared to that produced traditionally. Application of *L. plantarum* MNC 21 +  *S. cerevisiae* MNC 21Y starters in the modified process of *Enturire* fermentation can shorten the processing of *Enturire* from 5 to 7 days to only 12–24 hr thus making the commercial production of this product more feasible.

## Conflict of interest

No conflict of interest declared.
